# Economical Evaluation of Reduced Herbicide Doses Application Rates to Control *Phalaris brachystachys* (Short-Spiked Canary Grass) in a Biennial Wheat–Sunflower Rotation in Mediterranean Dryland: A Modelling Approach

**DOI:** 10.3390/plants13020212

**Published:** 2024-01-11

**Authors:** Casto Zambrano-Navea, Fernando Bastida, Maria J. Aguilera, Jose L. Gonzalez-Andujar

**Affiliations:** 1 Departamento de Agronomia, Facultad de Agronomia, Universidad Central de Venezuela, Maracay 4579, Venezuela; castorzambrano@gmail.com; 2 Departamento de Ciencias Agroforestales, Universidad de Huelva, 21007 Huelva, Spain; bastida@uhu.es; 3 Departamento de Fisica Aplicada, Universidad de Cordoba, 14071 Cordoba, Spain; fa1agurm@uco.es; 4 Instituto de Agricultura Sostenible (CSIC), 14004 Cordoba, Spain

**Keywords:** population dynamic, sensitivity analysis, weed control, weed competition, annualized return rate, bioeconomic model

## Abstract

*Phalaris brachystachys* (short-spiked canary grass) is considered to be among the most troublesome cereal weeds in Mediterranean areas. A bioeconomic model, based on population dynamics, competition and economic sub-models, was developed to simulate the long-term economic consequence of using herbicide-based strategies: no herbicide application, full herbicide dose (standard rate) and two reduced dose rates (75 and 50% of the standard rate) to control *P. brachystachys* in a biennial wheat–sunflower rotation. Simulation results indicated that only herbicide application at a full dose (90% control) and 3/4 dose (80% control) produced positive economic results, with the full dose being the best strategy (EUR 98.65 ha^−1^ year^−1^). A sensitivity analysis showed that the economic outcome, in terms of annualized net return, was strongly influenced by changes in yield, price, and fixed costs. In addition, the annualized net return was more sensitive to parameter changes at reduced herbicide doses than at full rate. In the wheat–sunflower rotation system, the application of the full dose of herbicide was the most economical and stable strategy in the long-term. Reduced doses are not a recommended option from an economic point of view. Bioeconomic models provide practical insight into different management approaches for effective weed control.

## 1. Introduction

Short-spiked canary grass (*Phalaris brachystachys* Link) is an annual grass that is one of the most problematic weeds in cereal cropping systems in Mediterranean regions [1,2] and is generally found in agricultural settings, pastures, and along roadsides. *P. brachystachys* thrives in fertile soils and requires a sufficient amount of soil moisture for optimal growth. It can tolerate a moderate to high level of moisture. The ideal soil texture for this species ranges from loamy-sandy to heavy clay. It is a major problem in cereals in Spain, especially in the south of the country. In a study by Gonzalez-Andujar & Saavedra [2], 17% of the fields surveyed were infested with this species.

*P. brachystachys* is a prolific seed producer and has been spread unintentionally by human activity and as a contaminant of agricultural products. The fecundity of *P. brachystachys* has been established at an average value of 1232 seeds plant^−1^, hence, high annual growth rate (76%) [3], indicating a high potential for population increase in the absence of control measures. Without treatment, the seed bank can potentially build up 54,859 seeds m^−2^ after 25 years and it can decrease wheat yield by 16% to 60%. Infestations of 152 and 304 plants m^−2^ can reduce wheat yield by 32% and 42%, respectively [4]. In Spain, 100 plants m^−2^ reduce wheat yield by 16% [1]. Although herbicides are widely used to control this species in wheat monocultures, it has been shown that herbicide tactics are not enough to keep short-spiked canary grass in check [5]. The difficulty in controlling *Phalaris* ssp. in wheat is due to the botanical and physiological proximity of the two species, which limits the efficacy and selectivity of herbicide active substances.

In southern Spain, the biennial sunflower–winter cereal rotation has been implemented during the last half century with the aim of increasing the use of resources and the control of winter weeds, currently forming a consolidated and stable agricultural system. The sunflower crop is managed under a low level of inputs exploiting their complementarity with cereal crop water and nitrogen use. This has been mainly attributed to the different root architecture of both crops, with the deeper sunflower roots reaching soil resources not accessed by the cereal roots. Winter cereal–sunflower rotation facilitates the breaking up of the weed cycle [1]. In sunflower years, populations of short-spiked canary grass is severely reduced by pre-sowing tillage operations at mid to end winter [1], whereas its control in wheat years is based on herbicide applications [6,7]. *P. brachystachys* can be controlled with different herbicides, i.e., diclofop, tralkoxydim, fenoxaprop [4]. However, the relatively high cost of these products and their negative environmental effects have led growers to consider the use of low herbicide doses. Some studies have indicated the potential for reducing herbicide doses by 25–40% without compromising herbicidal effectiveness or causing significant yield reduction [8,9]. Experimental studies conducted with *Phalaris* spp. in southern Spain have shown that reduced herbicide doses of clodinafop in the range of 50–75% exert enough control without reducing crop yield [10].

Mathematical modelling plays a crucial role in advancing ecological knowledge. By quantitatively and qualitatively describing ecological processes and interactions, these models allow ecologists to gain insights into the functioning of ecosystems, make predictions, and inform management and conservation strategies. By simulating different scenarios and considering various factors, these models provide valuable insights into the effectiveness of weed control programs and help guide sustainable management practices. Population dynamic models have been employed to explore the long-term impact of weed management tactics [11], including the application of low herbicide doses on *P. brachystachys* in wheat [5] and in wheat–sunflower rotation [12]. However, there is a lack of information on the economic implications of these approaches. If we are to provide useful advice to farmers, it is necessary to include some economic parameters in our models in order to assess the monetary implications of management strategies. In this context, the use of bioeconomic modelling is an appropriate tool to explore the long-term economic consequences of implementing weed management strategies.

The development and use of bioeconomic models in weed science have a long history, providing valuable insights and guidance for weed management decision-making. A bioeconomic model combines biological knowledge about weed growth, reproduction, and competition with economic analysis to make informed decisions regarding weed management strategies over the long term [13,14,15,16]. Therefore, the aim of this study was to develop a bioeconomic population-based model for *P. brachystachys* and employ it to make an economic assessment of the long-term impact of applying of reduced herbicide rates on controlling this species in a wheat–sunflower rotation.

## 2. Results

**Figure 1:**
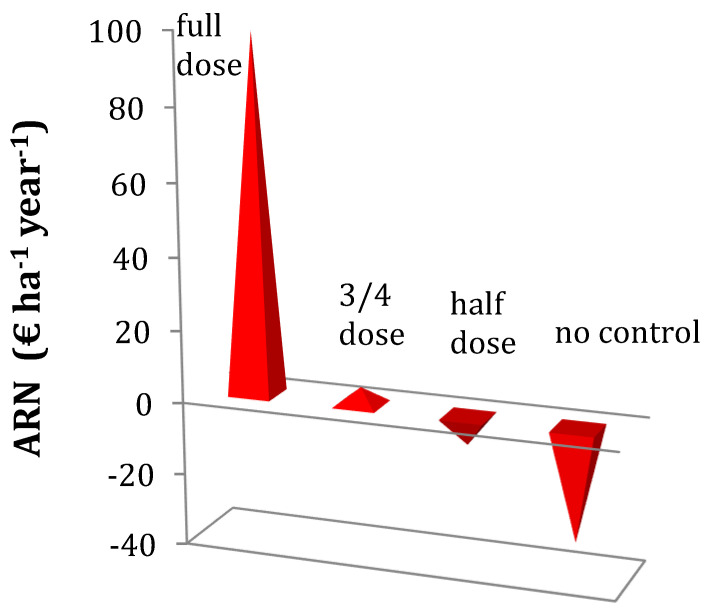
Annualized Net Return (ARN) of the wheat–sunflower rotation under different doses of the post-emergence herbicide clodinafop for the control of *Phalaris brachystachys* in wheat. The different doses and their corresponding control percentages are as follows: full dose (90% control), ¾ dose (80% control), half dose (70% control), and no control (0% control).

The full application rate of clodinafop (see Material and Methods) in the wheat year, as applied by the farmers, produced the best economic result measured by the annualized net return (ARN) (EUR 98.65 ha^−1^ year^−1^). However, when the rate was reduced by 25% (equivalent to 3/4 of the standard application rate), the economic result dropped significantly to EUR 4.80 ha^−1^ year^−1^. This corresponds to a strong economic reduction of 95.13% in profit (Figure 1).

Further reductions in the rates of application resulted in negative ARN, rendering it economically unfeasible. Specifically, reducing the dose by 50% led to a loss of EUR −7.93 ha^−1^ year^−1^ and, as expected, the complete elimination of the herbicide application resulted in the worst economic outcome at EUR −31.45 ha^−1^ year^−1^ (Figure 1).

The parameters are used as constants in the bioeconomic model (see Material and Methods), but in reality they experience variations that can alter the output of the model. To account for such a circumstance, we consider the use of a sensitivity analysis with a variation of ±40% in the economic parameter values. Overall, the sensitivity analysis showed that the ARN was highly sensitive to variations in yield, price, and fixed costs (Table 1). It is noteworthy that the ARN was more sensitive to the ¾ and half doses than to the full doses of herbicide application (Table 1).

## 3. Discussion

In this work, a bioeconomic model of *P. brachystachys* has been developed and used to simulate various herbicide-based strategies in a biennial wheat–sunflower rotation. The inclusion of an economic analysis was essential to test economically viable weed control strategies and present a more realistic approach to farming interests. Four control scenarios were simulated in order to obtain their economic output. Our results suggest that the application of full dose was clearly the most economically efficient strategy (EUR 98.65 ha^−1^ year^−1^; Figure 1). This result is similar to that obtained in a field experiment carried out by Garcia [17] to control *P. brachystachys* in wheat, where an average value of EUR 123.23 ha^−1^ was obtained with a full dose application of clodinafop. Not controlling this species leads to an economic loss of EUR −31.45 ha^−1^ year^−1^ (Figure 1) and the establishment of an important seed bank [12]. Previous results on this species indicate that effective population control can only be achieved by applying the full herbicide dose [4,12].

The application of reduced herbicide doses of clodinafop can decrease the population of *P. brachystachys* but is not effective enough to keep populations of *P. brachystachys* under control [5,10]. In addition, when reduced rates are used in the wheat year, they often fail to control weeds effectively, allowing some plants to survive. This survival can contribute to the establishment of a long-term seed bank, exacerbating the infestation. From an economic point of view, none of the reduced herbicide dose strategies gave good net returns; only the application of the ¾ dose generated low positive ARN values (EUR 4.81 ha^−1^), whereas the use of 50% of the recommended dose generated negative ARN values (EUR −7.93 ha^−1^) (Figure 1). These results agree with those presented by Roggenkamp et al. [18], who reported that the use of reduced doses of alachlor and atrazine in corn (Zea mays L.) for the control of velvetleaf (*Abutilon theophrasti*) and green foxtail (*Setaria viridis*) presented few economic benefits, and contradict those presented by Barroso et al. [19], who report that reduced herbicide doses produce good net yields in *Avena sterilis* in wheat and Barros et al. [20] who argue that the use of reduced doses of herbicides does not imply financial risk. In contrast with these authors, we found few possibilities for reducing the herbicide dose from an economic point of view, as only the application of 3/4 of the dose produced long term economic benefits, although it was associated with a high variability due to its high sensitivity to variations in the model parameters.

Gaitan et al. [10] have suggested the potential of using reduced clodinafop doses in combination with a wheat–sunflower rotation for controlling *Phalaris* spp. populations. Their findings suggest that this approach can be an effective short-term strategy, offering significant weed control of *P. brachystachys* while minimizing the environmental impacts associated with the herbicide use. However, our results suggest that reducing the standard rate of clodinafop application is not an economically efficient strategy in the long term. The application of the full dose was the most efficient and economically stable strategy in the long-term, whereas the use of reduced doses of herbicides is not recommended.

The primary weed control method used by farmers in cereal crops is the application of herbicides, which has been the main focus of this study. Nevertheless, reducing the herbicide dose might increase the potential for herbicide resistance development [21,22,23,24], especially in agroecosystems with high weed densities and adverse environmental conditions, such as those found in cereal crops in rainfed Mediterranean areas. In the latter, herbicide efficacy is not optimal, and they can lead to the development of resistant populations. In this regard, Diaz et al. [5], showed that the application of reduced doses of clodinafop was not able to reduce *P. brachystachys* populations in the long term, and, as a result, populations are gradually exposed to selective pressure and can develop resistance to the herbicide.

Golmohammadzadeh et al. [22] reported the first case of resistance due to both target sites of *P. brachystachys* to clodinafop (ACCase inhibitor). The use of herbicide dose reduction strategy has to be managed with criteria that take into account the potential risk associated with it. Alcantara et al. [7] demonstrated, for the *Phalaris* spp., that the efficacy of herbicides is higher when optimizing the timing of chemical control. Despite the support from numerous studies regarding on the efficacy of reduced herbicide doses, there remains skepticism about their long-term cost-effectiveness, particularly in situations with high weed density or unfavourable environmental conditions. Our study further demonstrates this skepticism in the context of a wheat–sunflower rotation, showing diminishing economic returns as herbicide doses are reduced. While our research focuses on the application of clodinafop, we hypothesize that similar results could be observed with other herbicides.

In this study, ARN was particularly sensitive to variations in potential yield, price and fixed costs. Other authors have found similar results in annual weed species infesting cereals. Gonzalez-Andujar and Fernandez-Quintanilla [25] for a bioconomic model of *Avena sterilis* growing in winter wheat found the most sensitive economic parameters were fixed costs and potential yield. Torra et al. [26], in a study on *Papaver rhoeas*, found that their model was particularly sensitive to grain yield and cereal price. In the study by Gonzalez-Diaz et al. [14], potential yield and wheat price were identified as the most important parameters in a bioeconomic model for the co-management of *Lolium rigidum* and *A. sterilis* in winter wheat. The marked difference in terms of sensitivity values of the ARN between full dose and reduced doses applications of clodinafop suggests a potential economic risk in the long-term use of the reduced herbicide rates. Full-rate applications showed greater stability to variations in economic parameters than the reduced-rate applications. Barroso et al. [19] pointed out that under adverse environmental conditions, half rates of the herbicide failed to control *Avena sterilis* ssp. *ludoviciana* populations adequately. Therefore, the marked difference in terms of sensitivity values between full-rate and reduced-rate applications suggests a potential economic risk in the long-term use of the reduced herbicide rates. For instance, the ANR values can be significantly affected by the variability of wheat and sunflower yields in Mediterranean agroecosystems [27,28,29]. The detailed study of these sensitive parameters under field conditions at different locations will facilitate the validation of the model under different conditions.

The main focus of this study has been the utilization of herbicides as the primary method of weed control for farmers in the wheat–sunflower rotation. However, frequent use of the same herbicide can lead to the emergence of resistant weed populations [30]. This resistance problem emphasizes the importance of adopting an integrated approach to weed control, which involves a combination of preventive, mechanical, chemical, and cultural measures. Cultural practices at planting and crop rotation are practices that can be effective in controlling *P. brachystachy*. For instance, the inclusion of a fallow year in the crop rotation has been shown highly effective in reducing *A. sterilis* populations in wheat in Spain. The use of competing crops and high seeding rates have also been shown to be effective weed control strategies. The integration of cultural management strategies with the application of low herbicide rates could provide an agronomically viable, profitable and environmentally friendly solution for the control of *P. brachystachy*.

Nevertheless, in order to effectively use bioeconomic models as decision-making tools for farmers, it is essential to validate the model in real-world scenarios [11]. Further development of the model should include validation and considerations of other management strategies, and the integration into Decision Support Systems [31] to provide farmers and crop advisors with decision tools in real-time. The study’s findings have important implications for farmers and agronomists seeking cost-effective methods to control *P. brachystachy* populations in cereal drylands and improve crop yields. There is a widely acknowledged necessity for the collaboration between ecology and economics disciplines in order to inform policy makers on the importance of effective weed management and provide them with economically viable methods of prevention and control for successful agricultural policies. Bioeconomic models offer valuable assistance in this regard. In this study, we have amalgamated biological, agronomic, and economic data to construct a bioeconomic model, which proves to be an effective approach in determining the comparative profitability of various control strategies. Furthermore, the modelling methodology used in this research could be extended to other annual plants with similar life history characteristics within a rotation system.

## 4. Materials and Methods

### 4.1. Bioeconomic Model Structure

The bioeconomic model integrates three sub-models: population dynamics, weed–crop competition and economic [13]. This study expands a previous population dynamics model for *P. brachystachys* [12], including weed–crop competition and an economic analysis.

#### 4.1.1. Population Dynamics Sub-model

**Figure 2:**
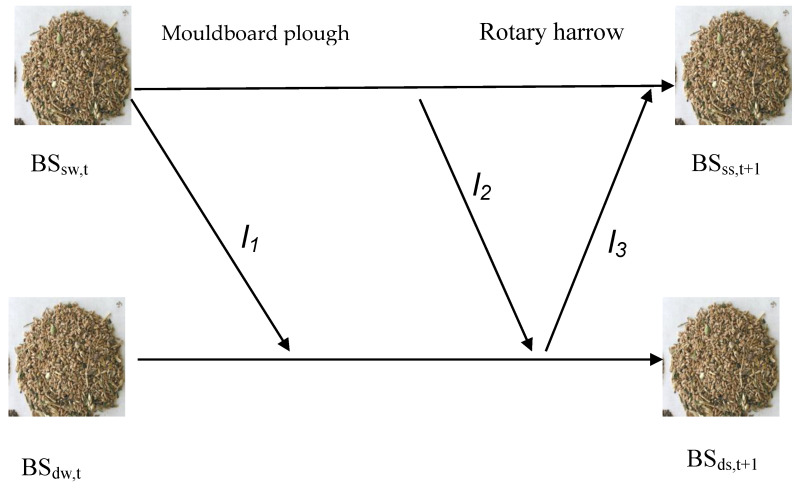
Seed movement of *Phalaris brachystachys* in the soil in sunflower crop. *l_1_*: rate of seed transfer to the deep seed bank. *l_2_*: rate of seeds incorporated to the deep seed bank. *l_3_*: rate of seeds incorporated to the shallow seed bank. BS_sw,t_: shallow seed bank in wheat. BS_dw,t_: deep seed bank in wheat. BS_ss,t+1_: shallow seed bank in sunflower. BS_ds,t+1_: deep seed bank in sunflower. *t* is time (Modified from Zambrano-Navea et al. [12]).

The population dynamics sub-model for *P. brachystachys* in the wheat–sunflower rotation has already been described [12]. It combines life-cycle population dynamics models for *P. brachystachys* during the wheat and sunflower growth stages [13,32] (Figure 2) and is described by the following mathematical structures:

- *P. brachystachys* population dynamics model in wheat

In brief, every year a proportion (*m*) of the seeds in the seed bank die naturally, while another portion (*g*) successfully emerges. The density of plants that grow and reach adulthood is denoted as *P_t_*. A fraction (*s*) of these plants survive until they can reproduce. On average, every surviving plant produce *f* viable seeds, which add to the seed supply that is stored in the seed bank. However, before integrating in the seed bank, a proportion *p* of the seed rain is lost due to biotic and abiotic factors (e.g., predation). The seed bank was split out into two soil layers: a deep seed bank (>5 cm) and a shallow seed bank (0 to 5 cm).

The shallow seedbank, i.e., the number of seeds that remain in the soil from the previous sunflower season, is modelled by the following:

*BS*_sw,*t+1*_ = (1 − *g*) (1 − *m*) *BS_s_*_w, *t*_ + *gsf* (1 − *p*)P_t_ (1)

The relationship between weed plant density and fecundity (density-dependence factor) can be expressed as follows:

*f* = *f*_0_/(1 + aP_t_) (2)

In this equation, *f₀* represents the number of seeds produced by a single, isolated weed plant. The parameter *a* represents the area required for a weed plant to produce *f₀* seeds. The density of the seed bank in the deep soil layer (*BS*_dw,*t*_) at time t is represented as follows:

*BS*_dw,*t+1*_ = (1 − *m*) *BS*_dw,*t*_ (3)

- *P. brachystachys* population dynamics model in sunflower

During a sunflower growing season, *P. brachystachys* does not yield any seeds. This is because all the plants are uprooted during tillage activities in mid- to late winter and seedbed preparation in late winter. Nonetheless, the seedbank is influenced by the vertical displacement of seeds in the soil caused by tillage operations. This movement occurs through the use of a moldboard plow followed by rotary harrow passes, ultimately affecting the population dynamics of *P. brachystachys.*

The shallow seedbank (*B_ss_*) is given by the following:

*BS*_*s*s, t+1_ = [*BS*_sw,*t*_ (1 − *l_1_*) (1 − *l_2_*) + *BS*_dw, *t*_
*l_3_*] *d_1_d_2_* (4)

l_1_ is the rate at which seeds move from the shallow to the deep seed bank after the moldboard plow pass. l_2_ is the rate of seed movement after the harrow pass. The rate at which seeds move from the deep to the shallow seed bank after the plow pass is denoted as l_3_. The survival rates of seeds after the plow and harrow passes are denoted as d_1_ and d_2_, respectively (Figure 2).

The deep seed bank in sunflower crop (*BS_ds_*) is as follows:

*BS_ds,t+1_* = [*BS*_dw,*t*_ (1 − *l_3_*) + *BS*_sw,*t*_
*l_1_ l_2_*] *d_1_d_2_* (5)

#### 4.1.2. Weed–Crop Competition Sub-Model

Weeds compete with crops for resources such as nutrients, water, light, and space, leading to reduced crop yield and quality. From a practical point of view, it is of utmost importance to be able to predict the effect of weeds on crop yields. To this end, empirical competition models have been developed that relate yield loss in response to weed density. The weed–crop competition sub-model has been based in the hyperbolic competition model [33], which establishes the functional relationship between the density of *P. brachystachys* and wheat yield, but the effect of competition on sunflower was not considered due to the absence of *P. brachystachys* in the sunflower year:

*Y_t_* = *Y_o_* ∗ (1 − (*b* ∗ *P_t_*/100 ∗ (1 + *b* ∗ *P_t_*/*a*))) (6)

where *Y_t_* (kg m^−2^) is the yield of wheat or sunflower in time t, *Y*_0_ (kg m^−2^) is the yield in the absence of weeds, *A* (adult plants m^−2^) is the density of *P. brachystachy* in time *t*, *a* and *b* are parameters. During the sunflower phase *A* = 0, and therefore *Y_t_* = *Y_o_*.

#### 4.1.3. Economic Sub-Model

This sub-model describes the economic impact of the different management scenarios considered on the net return (RN, EUR ha^−1^). In any year the NR is given as follows:

*NR_t_* = *P* ∗ *Y_t_* − *H* − *F* (7)

where, *Y_t_,* (kg ha^−1^) is the production of the crop at time *t*, *P* (EUR ha^−1^) is the crop price, *H* is the cost of herbicide and *F* (EUR ha^−1^) are fixed costs (seeds, fertilizers, etc.).

To facilitate the comparison of outcomes across various simulations, it was necessary to convert the net return over a specific duration of *n* years into the present-day currency [26]. Therefore, the annualized net return (ARN, EUR ha^−1^ year^−1^) was determined using the following expression:

*ARN* = (*Σ NR_t_* (1 + *i*)^−*t*^) (*i*/(1 − (1 + *i*)^−*t*^)) (8)

where, *i* is an annual discount factor, and *t* is time.

### 4.2. Models Parameterization and Initial Conditions

The value of the parameters was obtained from different available sources [5,10,34,35] (Table 2).

Initial seedbank was set at 100 seeds m^−2^, corresponding to a moderate infestation of about 16 seedlings m^−2^. The simulations were carried out over a span of 25 years, ensuring a sufficient duration to attain the population stability.

### 4.3. Control Strategies Considered

The control strategies considered were based on the use of the clodinafop (TOPIK 24^®^). This is a systemic post-emergence herbicide with no residual action for the control of certain grasses in wheat and triticale. It is absorbed by leaves and stems and works by inhibiting acetyl coenzyme carboxylase (ACCase). Once applied, the herbicide translocates via the phloem to the meristematic growth tissues and stops growth within two days, with clear effects of the herbicide being seen from the third week onwards.

Several individual tactics were simulated using different percentages of clodinafop application in wheat, namely 50%, 75%, and 100% of the standard rate. Tickes [36] found that using clodinafop (Topic24^®^, 250 mL ha^−1^) at the standard rate resulted in a 90% reduction in the *P. brachystachys* populations. In our own unpublished research, using the recommended rate of clodinafop (60 g ha^−1^ a.i.) at 50% and 75%, we observed reductions in the population of *P. brachystachys* of 70% and 80%, respectively.

### 4.4. Sensitivity Analysis

The model parameters have been assumed to be constant, but they may be subject to variations caused by various factors (e.g., temperature) that are likely to change their value from one year to the next. A sensitivity analysis was performed to account for potential variations in the parameters. This valuable analytical technique examines how different sources of uncertainty in a mathematical model contribute to the overall uncertainty of the model. The sensitivity index (ε) was used to assess ARN sensitivity [3,37,38] to herbicide application:

(9) $$\varepsilon =\frac{\left(\frac{f\left(p+∆p\right)}{f\left(p\right)}\right)}{\frac{∆p}{p}}$$

where, *p* is the parameter value, ∆*p* is a deviation from this value and *f (.)* represents the model output (ARN, EUR ha^−1^ year^−1^) after 25 years. A large value of ε indicates that the model is very sensitive to small variations in the parameters. Each parameter was subjected to +/−40% variation as being representative of the high variability in Mediterranean climate in rainfed annual crops [5,39].

## 5. Conclusions

The bioeconomic model developed here integrates a dynamic model of *P. brachystachys’* population growth, a competition model with the crop and an economic model. The model provides a comprehensive representation of the wheat–sunflower rotation characteristic of the Spanish cereal drylands. This type of model allows the assessment of the economic benefits of different management strategies and can help farmers and technicians to make informed decisions regarding weeds. In the long term, the simulation results showed that the use of full-rate herbicide application, compared to reduced-rate application, was a highly stable and economically efficient approach for effective control of *P. brachystachys* in a wheat–sunflower rotation.

In summary, the bioeconomic model presented in this study provides a useful tool for evaluating and economically optimizing *P. brachystachys* control strategies in the wheat–sunflower rotations. By considering the biological and agronomic characteristics of the weeds, and the economic implications of control methods, the model provides a comprehensive framework for evaluating the most cost-effective and sustainable approaches to managing *P. brachystachys* and other similar weed species.

## Figures and Tables

**Table 1:** Sensitivity of annualized net returns (ARN) to variation in the values of the model’s parameters for three different herbicide doses for *Phalaris brachystachys* control in a wheat–sunflower rotation system after 25-year simulation.

Parameter	Change in Parameter Value (%)	Sensitivity Coefficients		
		Full Dose (90% Control)	¾ Dose (80% Control)	Half Dose (70% Control)
Wheat yield	+40 −40	4.84 0.15	30.59 −24.55	−11.30 16.30
Sunflower yield	+40 −40	3.77 1.22	28.63 −23.63	−13.33 18.33
Wheat price	+40 −40	4.84 0.15	29.55 −24.55	−11.30 16.30
Sunflower price	+40 −40	3.77 1.22	28.63 −23.63	−13.33 18.33
Herbicide cost (Wheat)	+40 −40	1.18 1.81	−2.37 7.37	4.47 0.53
Wheat fixed costs	+40 −40	1.16 3.83	−14.86 12.86	19.08 14.08
Sunflower fixed costs	+40 −40	1.52 3.47	−17.43 12.44	14.58 −9.58

**Table 2:** Model parameter values obtained from the literature.

Parameters	Value	Units
**Life-cycle sub-model**		
*Wheat*		
Seedling emergence (*e*)	0.16	1
Seedling survivorship (*s*)	0.19	1
Survival of seeds after chisel plough (*d_1_*)	0.50	1
Fecundity (*f*)	1454	seeds plant^−1^
Seed entering the seedbank (*v*)	0.90	1
*Sunflower*		
Seed remaining at surface after mouldboard plough (*l_1_*)	0.03	1
Survival of seeds after mouldboard plough (*d_2_*)	0.75	1
Germinated seeds (*g*)	0.01	1
Seed remaining at surface after chisel plough (*l_2_*)	0.49	
Seed ascended to chisel plough (*l_3_*)	0.20	1
Survival of seeds after chisel plough (*d_1_*)	0.50	1
Herbicide control (*c*) at 100%, 75%, 50% and 0% recommend rate	0.90, 0.80, 0.70, 0	1
**Weed–wheat competition sub-model**		
Potential wheat yield	2800	kg ha^−1^
Potential sunflower yield	1000	kg ha^−1^
*A*	0.41	1
*B*	86.53	1
**Economic sub-model**		
Wheat price	0.18	EUR kg^−1^
Sunflower price	0.26	EUR kg^−1^
Wheat fixed costs	252	EUR ha^−1^
Sunflower fixed costs	200	EUR ha^−1^
Herbicide cost		
Full dose	60	EUR ha^−1^
¾ dose	45	EUR ha^−1^
Half dose	30	EUR ha^−1^
Control	0	
Annual discount factor	3	%

## Data Availability

The data is contained within the manuscript.
